# Advances in Gene Therapy for Xerostomia: Current Perspectives and Future Directions: A Narrative Review

**DOI:** 10.1002/hsr2.72295

**Published:** 2026-04-30

**Authors:** Muhammad Usman Bilal, Sarah Alvi, Fatima Humayune, Hasan Mujtaba, Shahzad Ahmad, Shazia Iqbal, Muhammad Farooq Umer

**Affiliations:** ^1^ School of Dentistry Shaheed Zulfiqar Ali Bhutto Medical University Islamabad Pakistan; ^2^ Sindh Institute of Oral Health Sciences Jinnah Sindh Medical University Karachi Pakistan; ^3^ Department of Oral Pathology, School of Dentistry Shaheed Zulfiqar Ali Bhutto Medical University Islamabad Pakistan; ^4^ Faculty of Medicine and Health Science The University of Buckingham Buckingham UK; ^5^ Department of Preventive Dental Sciences, College of Dentistry King Faisal University Hofuf Al‐Ahsa Saudi Arabia

**Keywords:** aquaporin‐1, CRISPR‐Cas9, gene therapy, salivary gland regeneration, xerostomia

## Abstract

**Background and Aims:**

Xerostomia, or dry mouth, significantly impairs quality of life and oral health, particularly in patients subjected to radiation therapy or suffering from systemic disorders. This narrative review summarizes the current evidence on the efficacy and safety of gene therapy interventions for xerostomia, exploring innovative approaches such as viral vector‐mediated delivery of aquaporin‐1, growth factor‐based strategies, and cutting‐edge CRISPR techniques.

**Methods:**

We conducted a comprehensive literature search on gene therapy interventions for xerostomia to synthesize this narrative review.

**Results and Conclusion:**

A critical evaluation of these studies reveals promising advancements alongside current limitations in medical research, including off‐site therapeutic effects, delivery efficiency, and long‐term efficacy. This review thus provides a comprehensive perspective on current gene therapy strategies for xerostomia, setting the stage for future research to bridge the gap between experimental therapies and everyday clinical applications.

## 1 Introduction

The salivary gland in humans is divided into major (parotid, submandibular, and sublingual) and minor salivary glands (hundreds of those in the oral cavity). The major glands contribute around 90% of the saliva secretion. The histology of salivary glands is well documented. The parts of the salivary gland that contribute to saliva are acinar cells and duct cells. Acinar cells occur at the culmination of complex, branched structures of ducts. Non‐epithelial entities, such as fibroblasts, macrophages, NK cells, Dendritic cells, and T cells, play a vital role in salivary glands [1].

Various proteins in acinar cells drive saliva formation, most crucially the aquaporins, specifically AQP1, AQP3, and AQP5 [2]. These proteins are crucial in the transfer of water molecules across the membrane. Another transport protein of interest concerned with electrolyte balance is NKCC1 [3]. The striated ducts maintain electrolyte balance and contain granules of kallikrein and glycoproteins. In the Excretory ducts, ionocytes release FGF10. CFTR in the ductal system participates in the resorption of NaCl and bicarbonate, thereby regulating salivary pH. This mechanism is absent in the murine salivary gland.

Secretion is initiated by activation of muscarinic receptors M1 or M3 by acetylcholine (ACh) or adrenergic receptors (α1) by norepinephrine (NE) and epinephrine (Epi) via specific pathways involving IP3 release or cAMP modulation. As a result of multifaceted, complex interactions across different channels and within cellular organelles, saliva composition is determined.

Saliva primarily helps with digestion and defense, as well as decreasing caries susceptibility through a repertoire of biomolecules [4, 5]. Local manifestations of salivary gland hypofunction may include dental erosions, halitosis, hypersensitivity, and others. Systemic Implications may consist of pharyngitis, laryngitis, and infections [6]. Clinically, Xerostomia is either described as subjective dryness in the mouth or the objective decreased saliva in the mouth. It may be due to impairment of the interplay among various secretory pathways [7]. Usually, the stimulated salivary flow rate is 1.5–2.0 mL/min, and the unstimulated salivary flow rate is 0.3–0.4 mL/min. Hyposalivation is diagnosed when the stimulated salivary flow rate is less than 0.7 mL/min, while the unstimulated salivary flow rate is 0.1 mL/min [8].

Current management focuses on symptomatic relief. Medications such as pilocarpine and cevimeline enhance saliva flow, while electrostimulation and acupuncture promote saliva flow. Emerging options include stem cell and gene therapy [6, 8]. One promising treatment modality is gene therapy. This intervention aims to target channels, regulate gene expression, and block unwanted pathways to restore salivary flow rate and salivary gland function. This narrative review aims to link the intervention to the gland's cellular makeup. It will also briefly outline the intervention's limitations, pros, and cons to provide a clear overview that benefits clinicians and researchers.

## 2 Methodology

This narrative review follows an approach to synthesize current evidence on gene therapy for xerostomia. A comprehensive literature search was conducted using the major databases PubMed and Google Scholar. The search strategy employed a combination of keywords and Boolean operators (“gene therapy” OR “genetic intervention”) AND (“xerostomia” OR “dry mouth”) AND (“salivary gland” OR “treatment”) targeting studies published from 1997 to 2025. Inclusion criteria focused on primary studies and clinical trials specifically addressing gene therapy approaches for xerostomia, while studies unrelated or lacking apparent efficacy and safety outcomes were excluded.Any studies in any language other than English or published more than 35 years ago were excluded. Screening involved an initial title/abstract review, a full‐text assessment of relevant studies, and the final inclusion of 31 articles spanning in vivo, preclinical models (rodents, primates, swine), and human trials. Methodological quality was critically analyzed, noting common limitations in preclinical studies (e.g., small sample sizes, lack of blinding) and clinical trials (e.g., limited long‐term follow‐up).Acknowledged limitations included publication bias, database search, and variability in study designs. No ethical approval or consent was required as the study is a narrative review.

## 3 Pathophysiology of Xerostomia and the Components Involved That Can be Used for Gene Therapy

Xerostomia can have multiple causes, such as medications, radiation therapy, systemic diseases, and Sjogren syndrome [5]. Among these, the most discussed in the literature are Sjögren syndrome and salivary gland damage due to irradiation (IR).

### 3.1 Autosomal Dominant Aplasia of the Lacrimal and Salivary Glands (ALSG)

A mutation of *FGF10* gene can cause ALSG, leading to fewer or no salivary gland cells and xerostomia. This can be caused by a heterozygous mutation in FGFR2 or FGFR3; thus, these genes become crucial in our story [9].

### 3.2 Cystic Fibrosis

Patients with cystic fibrosis may also see an altered salivary electrolyte and protein composition because of a mutation in the *CFTR* gene. This condition not only affects salivary glands but also a host of other processes in the human body as well [10].

### 3.3 Mutation in NkCC1 Gene

Mutations in gene encoding for NKCC1, a sodium‐dependent chloride transporter, also lead to xerostomia as its primary role is providing the passage of Cl ions across acinar cells, thus regulating the composition of saliva [11].

### 3.4 Sjogren Syndrome

Xerostomia in Sjögren's syndrome results from an aberrant immune response targeting the salivary glands, leading to dry mouth. Initially, B‐cells produce autoantibodies against Ro/SSA, La/SSB, the M₃ muscarinic receptor, nuclear antigens (ANAs), and rheumatoid factor. The infiltrates that form around ducts and vessels are sustained by B‐cell activating factor. These infiltrates are also rich in CD4 cells (Th1, Th2, Th17, and T follicular helper (Tfh) subsets). Th17 cells secrete IL‐17, IL‐22, and IL‐21, which promote inflammation, while Tfh cells support the production of autoantibodies. A host of autoimmune reactions intensifies the insult. T cells also release IL‐6, IFN‐γ, TNF, and TGF‐β. Additionally, increased LAMP3 production induces apoptosis and degrades NKCC1 or AQP5, which are critical for saliva production. Thus, in SS, the interplay of autoantibody production, T‐cell–driven cytokine release, epithelial cell dysfunction, and progenitor cell senescence culminates in glandular destruction and profound hyposalivation. Understanding these molecular and cellular circuits highlights potential therapeutic targets [12].

### 3.5 Salivary Gland Irradiation

Despite advances in radiotherapy, over 80% of head and neck cancer patients experience salivary hypofunction, with a vast majority of them developing chronic or permanent loss. This results from radiation‐induced depletion of acinar cells, which fail to regenerate. Although the exact mechanisms are unclear, damage to the glandular microenvironment likely disrupts cell–cell signaling critical for secretion and regeneration. Acute hyposalivation results from a combination of DNA damage, acinar cell death, increased reactive oxygen species (ROS) production, and disruption of Ca2+ signaling. A decrease in MVD is also observed in the early days of salivary gland irradiation.

Long‐term dysfunction arises not only from acinar cell loss but also from damage to the surrounding microenvironment, including nerves, vasculature, and extracellular matrix (ECM). Irradiation (IR) disrupts parasympathetic innervation, reduces neurotrophic factors (e.g., NRTN, BDNF), and leads to sympathetic hyperinnervation. Endothelial cells are also damaged early post‐IR, impairing blood flow and VEGF expression. These changes hinder regeneration by disrupting critical cell–cell communication.

### 3.6 Gene Therapy as a Treatment Modality for Xerostomia

The application of gene therapy to salivary glands began in 1994 with the first report of vector‐mediated gene transfer in a mammal. This foundational work paved the way for translational research efforts aimed at restoring the salivary gland function [13].

Ongoing clinical trials and research efforts highlight the potential for gene therapy to become a practical treatment, motivating clinicians and researchers to pursue further advancements [14, 15, 16, 17]. Viral vectors can carry and deliver genes (whose expression is desired) into host cells. Non‐viral vectors can deliver genes into cells, such as plasmids (also called naked DNA), liposomes, or nanoparticles, to achieve the desired expression or result. Physical methods facilitate the uptake of genes into cells, such as electroporation, microinjection, or ultrasound‐mediated insertion [18]. CRISPR technology has also been used in this regard.

## 4 Viral Methods

### 4.1 Use of Adenovirus Vectors

#### 4.1.1 hAQP1‐Based Interventions Using Adenoviruses

Aquaporin‐1 (AQP1) gene therapy, one of the earliest strategies, involves introducing a transmembrane water channel to restore water transport in irradiated salivary glands [19].

Initial proof‐of‐concept studies using adenoviral vectors established the biological feasibility of this approach. AdhAQP‐1 was introduced into the ductal cells (which typically survive radiation therapy), followed by a single radiation exposure. One such study, using AdhAQP‐1in 1997 [20], showed a 2‐ to 3‐fold increase in salivary flow in mice. Another study demonstrated that AQP1 expression restored salivary secretion in irradiated mice to near‐control levels by enhancing acinar cell water permeability [21].

Different vectors, such as AAV‐2, were also tested to deliver hAQP‐1 (thought to have a broad host range, fewer associated diseases, and longer sustained expression), which resulted in a threefold increase in transepithelial water flow [22].

Subsequent studies experimented with larger models. Post‐Irradiation (10 Gy), monkeys were subjected to varying doses of AdhAQP‐1. Only the monkeys receiving low doses of AdhAQP‐1 had a markedly greater increase in salivary flow (one had a twofold increase and the other a 20%–50% increase) [23]. Another such study on larger models (miniature pigs) reported an 80% increase post‐third‐day IR (20 Gy) [24]. Follow‐up with an AAV‐2 vector encoding hAQP‐1 showed 35% recovery of salivary gland flow rate under a similar experimental design [25].

Additional preclinical work further characterized both efficacy and safety across species. Baum et al. evaluated Ad5‐mediated hAQP1 delivery in irradiated rats and miniature pigs and reported increased salivary flow without fatal adverse effects [26]. Follow‐up of this study showed an increase of 70%–500% in parotid flow 3–4.7 years post‐treatment [27].

A recent study showcased compensatory delivery of AQP1 via AAV‐2 vectors. This restored salivary secretion in LAMP3‐overexpressing (overexpression of LAMP3 promotes degradation of key salivary proteins, including AQP5, leading to reduced saliva production) mice, supporting the concept that AQP1 can functionally substitute for disrupted native aquaporin pathways [28].

Collectively, these preclinical advances have ultimately led to early‐phase clinical evaluation, including a Phase I trial using AAV2‐hAQP1, representing a critical step toward clinical translation of gene therapy for xerostomia [29].

#### 4.1.2 FGF and Vascular Endothelial Growth Factor‐Based Intervention

The potential radioprotective roles of adenoviral vectors carrying basic fibroblast growth factor (AdFGF) and vascular endothelial growth factor (AdVEGF) were observed in a 2007 study by Cotrim et al. (USA), which explored the role of endothelial cells in radiation‐induced damage to salivary glands. Pre‐treatment with AdbFGF or AdVEGF (IR‐treated mice) preserved MVD to a reasonable extent and, after 8 weeks, retained 70% and 80% of normal flow, respectively [30]. Similarly, a 2014 study by Guo et al. showed the potential of FGF2 gene therapy to reduce salivary dysfunction following fractionated irradiation (7.5 or 9 Gy over 5 days) in minipigs (7). A single pre‐administration of an AdLTR2EF1α‐FGF2 (an adenoviral vector designed to express the FGF2 gene under the EF1α promoter with LTR regulatory elements) protected microvascular endothelial cells and significantly limited the decline in salivary flow [31].

#### 4.1.3 Shh Gene Transfer Through Adenovirus Vectors

The *Shh* gene is involved in the triggering of the hedgehog pathway, which signals gland morphogenesis. This might help preserve salivary gland structure. To test this, the *Shh* gene was introduced into mice. The study found the intervention maintained nerve function by inducing neurotrophic factors and reduced vascular damage by promoting angiogenic factors, suggesting that transient hedgehog pathway activation is a promising strategy [32]. Similarly, pigs receiving a 20 Gy single‐dose irradiation to the right parotid gland, followed by *Shh* or control *GFP* gene delivery, showed preservation of acinar cells, parasympathetic innervation, and microvessels. This improved the stimulated saliva secretion up to 4 weeks [33].

#### 4.1.4 Adenovirus‐Mediated Neurturin Expression

In 2018, Ferreira et al. (USA) investigated whether a neurturin‐carrying adenovirus could be used for gene therapy. A Neurturin‐laden adenovirus was administered to mouse salivary glands. After a day, the salivary glands were irradiated. They were compared with the control group. The mice whose salivary glands were pre‐treated with neurturin were analyzed and showed results similar to those of non‐IR salivary glands, likely due to protection of the parasympathetic nervous system [34].

In a 2020 study by Lombaert et al. in the USA, the researchers used an Adeno‐associated virus serotype 2 (AAV2) vector carrying the human neurotrophic factor neurturin (CERE‐120). The researchers found that when the CERE‐120 was administered pre‐irradiation, salivary glands functioned normally in a mini‐pig model [35].

#### 4.1.5 Therapeutic Use of IL‐17 Using Adenovirus‐Mediated Gene Transfer

Recent studies highlight IL‐17A's role in primary Sjögren's syndrome. Its reduction in non‐susceptible mice suggests a key role in salivary gland dysfunction, making localized anti‐IL‐17 therapy a promising preventive strategy [36]. Nguyen et al. in 2011 in the USA experimented with targeting IL‐17 by blocking it (using an Ad5 vector). The results indicated success in decreasing the IL‐17 levels up to 18 weeks [37].

#### 4.1.6 Use of IL‐27

IL‐27 is a natural blocker of T_H_17 cells. These cells produce IL‐17. As mentioned previously, this IL‐17 is an instrument of Sjogren syndrome pathology. Lee et al. (2012, USA) used AAV2 vectors encoding IL‐27 or LacZ (control) in a study. Mice were used as specimens. The mice injected with rAAV2‐*IL27* showed long‐term elevation in serum IL‐27 levels. They also showed a decrease in IL‐17. These findings coincided with enhanced clinical findings of saliva flow rates. Leading authors to conclude IL‐27 administration to be a potential therapy for Sjogren syndrome [38].

#### 4.1.7 Use of IL‐10

IL‐10 is an anti‐inflammatory with significant therapeutic potential. Yamano et al. in the USA constructed a recombinant adeno‐associated virus serotype 2 vector encoding human interleukin 10 (rAAVhIL10). The vector was introduced into the mice's submandibular glands. Saliva was collected at different time points to assess IL‐10 concentration. The results showed a promising step forward in gene therapeutics [39].

Kok et al. performed an in vivo study using a submandibular model in rats. They engineered recombinant adeno‐associated virus (rAAV) vectors encoding human interleukin‐10 (rAAVhIL‐10) and administered them to irradiated mice. At 20 weeks post‐treatment, both the early and late rAAVhIL‐10 groups exhibited significantly higher salivary flow rates compared to the rAAVLacZ‐treated and untreated control groups [40].

#### 4.1.8 Use of Recombinant AAV‐9 TLK1b

As discussed above, TLK1B was also shown to protect salivary glands. Post irradiation (16 Gy), the TLK1B‐treated group experienced a 40% decline in salivary function at 8 weeks, compared to a 67% decline in the saline‐treated controls [41]. Shanmugam et al. investigated the use of AAV2/9 to deliver TLK1B to protect salivary glands from radiation‐induced xerostomia. TLK1B was retroductally administered to rat submandibular glands before fractionated irradiation (20 Gy). Results showed that TLK1B‐treated rats maintained normal salivary flow, with a 121.5% increase in stimulated output compared with > 90% reductions in the saline and control groups. Histological analysis revealed better preservation of gland structure, with reduced fibrosis and inflammation in treated glands [42].

#### 4.1.9 Adenovirus‐Mediated Stim1‐mKO1 Gene Transfer (Ad‐mKO1 or Ad‐Stim1mKO1) to Rat Submandibular Acinar Cells In Vivo

Monita et al. conducted a Japanese study in 2013 to investigate the potential of gene therapy to enhance calcium signaling and improve salivary secretion in rat submandibular glands. Researchers used an adenovirus vector to deliver a gene encoding Stim1‐mKO1, a calcium‐regulatory fusion protein, via retrograde ductal injection. The gene transfer led to a significant increase in calcium release and entry into salivary acinar cells, particularly during weak muscarinic stimulation [43].

To conclude, the literature on gene therapy for xerostomia using viral vectors has been a relatively well‐researched area in this domain. These studies are categorized with their stated outcomes in Table 1.

**Table 1:** A compendium showcasing the viral‐mediated gene therapy to mitigate hyposalivation.

Author/year hAQP‐1	Study design	Specimen	Method	Outcome
Delporte et al. 1997 [20]	In vivo experimental study	Rat submandibular glands	Retrograde ductal instillation of AdhAQP‐1 by viral vector	2–3‐fold increase in salivary secretion after 4 months
Braddon et al. study 1998 [22]	In vivo experimental study	Rat salivary glands	AQP‐1 protein was introduced into the salivary glands of mice by rAAV.	Transepithelial fluid movement is enhanced about threefold above basal levels.
O'Connell et al. 1999 [23]	In vivo experimental study	Adult rhesus monkeys	AdhAQP1 was administered to irradiated glands and to contralateral non irradiated glands	Did not result in a consistent positive effect on salivary flow rates in‐vivo
Zheng et al. 2001 [44]	In vivo experimental study	Wistar rats	Observing expression of hAQP1 after AdKALL‐hAQP1 gene transfer was observed in ductal cells in vivo	Delivering hAQP1 via the KLK1 promoter can restore salivary flow in irradiated rats.
Shan et al. 2005 [24]	In vivo experimental study	Miniature pig	Evaluated AdhAQP1‐mediated gene transfer after parotid gland IR in the miniature pig	Resulted in an increase in parotid salivary flow to ∼80% of pre‐IR levels
Goa et al. 2011 [25]	In vivo experimental study	Miniature pig	Irradiated parotid glands (20 Gy) were injected with AAV2‐hAQP1 and salivary flow and hAQP1 expression were monitored.	AAV2‐hAQP1 restored flow from ~15% to ~35% of normal, with hAQP1 localized to duct cells
Baum et al. 2012 [26]	Clinical trial	Human subjects	Dose of Ad5 carrying hAQP1 cDNA irradiated parotid gland of head/neck cancer survivors	Six out of eleven showed up to 540 percent increase, others showed no change
Teos et al. 2016 [21]	In vivo experiment	Mice subjects	Irradiated mice with reduced salivary flow received adenoviral hAQP1.	Restoration of water permeability, and restored salivary volume
Alevizos et al. 2017 [27]	Clinical trial	Human subjects	AdhAQP‐1 administered patients were followed for 3–4 years	Long‐term follow‐up showed sustained salivary flow increases.
Nakamura et al. 2022 [28]	In vivo experimental study	Mice	AAV2 vectors encoding AQP1 were administered via retrograde cannulation into salivary glands	AQP1 expression restored salivary flow in LAMP3‐overexpressing mice.
FGF and VEGF				
Cotrim et al. 2007 [30]	In vivo study	Mice	Administration of micro vessel protectors to prevent irradiation‐induced salivary hypofunction.	Micro vessel protection preserved salivary gland function post‐irradiation.
Guo et al. 2014 [31]	In vivo study	Mini pigs	Pre‐IR adenoviral FGF2 delivery to one parotid gland, followed by 5‐day fractionated IR, with salivary and blood flow monitored.	Protected microvascular endothelial cells, significantly preserved salivary flow,
Shh gene				
Bo Hai et al. 2016 [32]	In vivo study	Mice	Adenoviral Shh gene transfer via retrograde ductal instillation post‐irradiation, with assessment of its effects	Shh gene delivery activated hedgehog signaling, improved saliva secretion
Hu et al. 2018 [33]	In vivo study	Mini pigs	Single‐dose (20 Gy) irradiation of the right parotid, followed by adenoviral Shh or GFP gene transfer via retrograde ductal	Shh gene delivery (4 weeks post‐IR) improved saliva secretion and acinar cell preservation.
Ad neurturin				
Ferreira et al. 2018 [34]	In vivo and ex vivo	Mice and fetal salivary gland cultures	Neurturin‐expressing adenovirus was delivered 24 h before irradiation	IR‐induced hypofunction reduced and maintained normal salivary flow.
Lombaert et al. 2020 [35]	In vivo study	Mini pigs	AAV2‐neurturin (CERE‐120) was delivered pre‐ or post‐IR to submandibular glands, followed by immunostaining analysis.	Pre‐IR CERE‐120 prevented hypofunction, reduced fibrosis and immune responses, improved secretion
IL‐17				
Nguyen et al. 2011 [37]	In vivo study	Mice	Ad5‐IL17R: Fc were delivered to salivary glands to block IL‐17A signaling at pre‐disease or disease stages.	IL‐17A blockade increased saliva secretion and prevented SS progression.
IL‐27				
Lee et al. 2012 [38]	In vivo study	Mice	rAAV2‐IL27 vectors were injected into mice.	Reduced IL‐17, altered autoimmune responses, improved saliva flow
IL‐10				
Yamano et al. 2002 [39]	In vivo experimental study	Rat submandibular glands	rAAVhIL10 was injected into irradiated mice	hIL‐10 secretion into the was increased bloodstream
Kok et al. 2004 [40]	In vivo experimental study	Rats submandibular	rAAVhIL‐10 was introduced in irradiated mice	Salivary flow rates at 20 weeks were significantly higher than untreated mice.
Tousled‐like kinase 1B (TLK1B)				
Palaniyandi et al. 2011 [41]	In vivo experimental study	Rat submandibular salivary glands	Adenoviral delivery of TLK1B	After 16 Gy, TLK1B‐treated animals showed a 40% decline, versus 67% in controls.
Shanmugam et al. 2013 [42]	In vivo experimental study	Rat submandibular glands	AAV2/9 vectors with TLK1B or GFP were delivered retroductally to rat submandibular glands before fractionated radiation (20 Gy in 8 fractions).	Preservation of salivary flow post‐irradiation (121.5% increase), and gland histology was better maintained.
Stim1‐mKO1 gene transfer				
Monita et al. 2013 [43]	In vivo experimental study	Rat submandibular glands	Adenovirus administered to express Stim1‐mKO1 to submandibular glands	Increase muscarinic stimulation‐induced Ca (2 + ) responses in acinar cells

Abbreviations: AAV2, adeno‐associated virus serotype 2; AdhAQP1, adenovirus mediated human aquaporin‐1; Ad5‐IL17R:Fc, adenovirus serotype 5 interleukin‐17 receptor: Fc fusion viral vector; AQP, aquaporin; FGF 2, fibroblast growth factor 2; hAQP‐1, human aquaporin‐1; GFP, green fluorescent protein; IL‐10, interleukin‐10; IL‐17, interleukin‐17; IL‐27, interleukin‐27; IR, irradiation; LAMP3, lysosome‐associated membrane glycoprotein 3; rAAV, recombinant adeno‐associated virus; rAAVhIL‐10, rAAV vectors encoding human interleukin 10; rAAV2‐IL27, recombinant adeno‐associated virus serotype 2 interleukin‐27 vector; VEGF, vascular endothelial growth factor.

## 5 Non‐Viral Methods

Several non‐viral gene delivery techniques exist. Electroporation involves applying brief electrical pulses to open cell membranes, while others employ physical methods such as a gene gun, which propels tiny DNA‐coated particles into cells. Alternatively, genes can be delivered by directly injecting plasmid DNA. This DNA can be administered on its own as a “naked plasmid” or combined with positively charged lipids to form a “lipoplex.” Both methods have been successfully used to target salivary glands via intraductal cannulation [45, 46]. Some impactful studies regarding the non‐viral techniques have been categorized in Table 2.

**Table 2:** Tabulation showcasing the non‐viral mediated gene therapy to mitigate hyposalivation.

Author/year	Study design	Specimen	Method	Outcome
Epperly et al. 2004 [47]	In vivo experimental study	Mice	IR group of mice treated with single or multiple administrations of intraoral MnSOD‐PL	Mice treated with (MnSOD‐PL) showed a significant decrease in xerostomia
Passineau et al. 2010 [48]	In vivo	Rat submandibular glands	UAGT using CMV‐Luc plasmid was delivered to the submandibular gland. Gene expression was monitored	UAGT resulted in robust and stable gene expression for 28 days.
Szilvia Arany et al. 2013 [49]	In vitro	Mice	Nanoparticle‐mediated gene silencing confers radioprotection to salivary glands in vivo	Marked improvement in saliva secretion at 3 months in irradiated animals.
Wu et al. 2015 [50]	In vitro	Mice	Gene transfer was employed to induce expression of a fusion protein in the submandibular glands.	Non‐viral IL‐17 sequestration gene therapy in the salivary gland downregulates expression of a putative SS autoantigen.
Wang et al. 2015 [51]	In vivo experimental study	Mini pig	UAGT of AQP1 to the irradiated minipig parotid gland restores fluid secretion	UAGT can alternate the Adenoviral vector as a means of delivering AQP1 gene therapy in the irradiated swine model

Abbreviations: AQP‐1, aquaporin‐1; CMV Luc, cytomegalovirus promoter drives the expression of the luciferase reporter gene; Fc, fragment crystallizable region; IL‐17, interleukin‐17; IR, irradiated; MnSOD‐Pl, manganese superoxide dismutase‐plasmid/liposome; SS, Sjogren syndrome; UAGT, ultrasound‐assisted gene transfer.

Epperly and colleagues (2004) found that at higher doses (22.5–30 Gy), MnSOD‐PL protected against oral cell death and dry mouth by maintaining saliva output. Overall, the combination of MnSOD‐PL and WR272 (a radioprotective agent) significantly lessened oral damage and improved survival [47].

In a 2010 study conducted by Passineau et al. (USA), ultrasound‐induced microbubble destruction was explored for non‐viral gene delivery to mouse salivary glands. The CMV‐Luc plasmid was delivered to the salivary gland in a carrier solution. Out of the two solutions delivered non virally 15% microbubble solution enabled stable gene expression for 28 days, making it a potential method for ultrasound gene transfer to salivary glands [48].

Arany et al. (2013, USA) used pH‐responsive nanoparticles loaded with siRNAs to modulate gene expression in submandibular glands via retrograde injection. Targeting Nkcc1 reduced saliva production, mimicking systemic knockout effects. siRNAs against Pkcδ, administered pre‐radiation, reduced apoptosis and improved saliva secretion after 3 months [49].

A 2015 study by Wu et al. (USA) investigated the role of interleukin‐17 (IL‐17) in Sjögren's syndrome (SS) by employing ultrasound‐assisted non‐viral gene transfer (UAGT) to sequester IL‐17 in the salivary glands of SS mice. This approach successfully reduced both local and systemic IL‐17 levels. These findings suggest that targeting IL‐17 through gene therapy in the salivary glands is feasible and may downregulate proteins associated with SS pathogenesis [50].

A 2015 study by Wang et al. used an in vivo experimental minipig model. They demonstrated that UAGT of the AQP1 gene into the IR parotid gland effectively restored fluid secretion [51].

## 6 Use of CRISPR Technology

CRISPR‐Cas9 is a site‐specific gene editing technology that has revolutionized the field of gene therapy. The CRISPR‐Cas9 system has FDA‐approved clinical applications, including SARS‐CoV‐2 detection, sickle cell disease, β‐thalassemia, Duchenne muscle disorder, and many more [52]. CRISPR in xerostomia (Table 3) has been under‐researched compared to other medical applications.

**Table 3:** Tabulated overview of CRISPR‐Cas9 and dCas9‐SAM applications in gene therapy for xerostomia.

Author/year	Study design	CRISPR	Method	Outcome
Wang et al. 2017 [53]	In vitro	CRISPR‐Cas9	This study employed CRISPR‐Cas9 gene editing with the endogenous AQP1 promoter in HEK293 and MDCK cell lines.	Resulted in an increased transmembrane fluid flux, indicating improved salivary gland function
Wang et al. 2017 [13]	Human salivary ductal cell line (A253) and human stem/progenitor cells (hS/PCs).	Dead Cas9‐synergistic activation mediator (dCas9‐SAM	dCas9‐SAM and epigenetic editing were used to induce AQP1 expression by activating the native gene locus.	AQP1 expression was induced, highlighting epigenetic editing's potential for gene therapy.
Guan et al. 2024 [54]	In vivo	Mouse model	Radiation exposure and CRISPR activation of Tert in human SMG spheres.	Tert‐expressing cells led to salivary gland homeostasis and tissue regeneration post‐irradiation.

Abbreviations: AQP1, aquaporin 1; CRISPR‐Cas9, clustered regularly interspaced short palindromic repeats‐CRISPR‐associated protein 9; HEK293, human embryonic kidney 293; MDCK, Madin‐Darby canine kidney; dCas9‐SAM, dead Cas9‐synergistic activation mediator; hS/PCs, human stem/progenitor cells; SMG, submandibular gland; Tert, telomerase reverse transcriptase.

CRISPR was used to enhance native AQP‐1 expression (Wang et al. 2017). They evaluated the results of the CRISPR‐Cas9 gene‐editing system after irradiation‐induced xerostomia. The results showed overexpression of AQP1 protein, which, in turn, indicates intracellular fluid movement and represents a potentially viable method for treating xerostomia and associated conditions [53].

Similarly, Wang et al. used Dead Cas9‐Synergistic Activation Mediator (dCas9‐SAM), a modified version of the CRISPR‐Cas9 system. It was observed that the system upregulates the AQP1 gene in the salivary ductal cell line A253, with a slightly variable, but not clinically relevant, difference observed in the other salivary ductal cell line [13].

Guan et al. in the USA identified a telomerase‐expressing (TertHigh) cell population in the ductal cells of the submandibular gland. The function of these TertHigh cells is to support the submandibular gland. These cells demonstrate an excellent ability to regenerate after radiotherapy, and CRISPR‐activated Tert in the human submandibular gland regenerates increasingly after radiation. The study suggests that increasing the expression of TertHigh cells by CRISPR can be beneficial in creating a hedge against radiation [54].

There is still no clear verdict on the long‐term safety and efficacy of the CRISPR‐Cas9 system, especially for its use in xerostomia. Potential side effects of the CRISPR‐Cas9 system have also been reported, including off‐target effects, reduced therapeutic efficiency in some cases, limitations of the Cas9 protein, and debate over the safety of CRISPR and gene editing [53].

## 7 Comparative Analysis of Viral, Non‐Viral, and CRISPR Methods

Among the various gene therapy strategies explored for xerostomia, viral vector‐based delivery remains effective to date. Viral vectors have demonstrated robust restoration of salivary flow in preclinical trials, showing relative yet not sufficiently long‐term improvement. Their major limitation lies in immunogenicity and restricted repeat dosing, particularly with adenovirus, though localized ductal administration reduces systemic risk.

Non‐viral methods (as listed above) offer a safer, repeatable alternative, achieving meaningful but short‐lived expression and radioprotective effects in animal models. These approaches are especially promising for prophylactic use before radiotherapy, where multiple administrations may be needed. The low immunogenicity would entail lower costs, as regular monitoring and follow‐up for safety could be spared compared to viral gene therapy. In contrast, CRISPR‐based platforms represent a newer paradigm, enabling direct editing or activation of endogenous genes such as AQP1 or TERT to promote intrinsic regeneration rather than transient expression. CRISPR approaches still face significant hurdles in targeted delivery, off‐target editing, and safety validation before translation into humans.

Overall, viral vectors currently provide the most substantial therapeutic impact for post‐radiation xerostomia, non‐viral systems prioritize safety and reusability, and CRISPR technologies chart a future direction toward permanent regenerative cures. Salivary glands are accessible by retrograde ductal cannulation. That favors a high local dose with limited systemic exposure. It is an advantage shared by all three approaches. All these therapies are still far from suitable for clinical use, as no long‐term expression of the desirable proteins or genes has been observed. These therapies also face a lack of robust clinical trials and regulatory hurdles to genetic modifications in humans.

## 8 Limitations and Future Directions

Gene therapy has the potential to provide a novel and effective treatment for xerostomia, but it also has some challenges and limitations. Translation from the laboratory to the everyday clinic has been slow due to broader challenges in gene therapy. Limited numbers of research groups focusing on this area, and gaps in understanding fundamental salivary gland biology, such as the exact nature and pathways involved in radiation‐induced salivary gland damage, so more appropriate and precise clinical therapies can be stipulated [55, 56, 57]. No robust evidence was found for long‐lasting effects of the treatments on the salivary glands, which are essential, as loss of salivary flow rate due to radiation is a long‐term problem. There is concern about toxicity and the potential to trigger an unfavorable immune response, as seen in some studies [27, 58, 59]. These downsides might be solved by experimenting with more efficient, less immunogenic vectors [60, 61].

Another limitation is the use of single‐dose radiation in many studies, which is not representative of how management of head and neck cancer is usually undertaken, that is, by fractionated radiation doses. Specimens such as mice, rodents, pigs, and primates may not replicate human immune responses or respond to interventions in the same way, and thus do not fully reflect the performance and applicability of gene therapy for xerostomia in the real world [62].

Lastly, there are significant regulatory hurdles that are required for these therapies to be used in everyday life, which may not reflect the clinical reality with the scientific advancement [63].

Future trials must also develop metrics to evaluate the proteomic quality of restored saliva, not just the volume. Success should be defined by the restoration of the salivary proteome, which is necessary to maintain oral homeostasis. A combination of these therapies may also be used to reap their benefits, a potential area for exploration. Larger multicenter studies would pave the way for faster adoption. Further research and development into recent advances, such as CRISPR, nanoparticles, and other non‐viral modalities, should be the central focus. These arenas have the potential to make a robust, clinically effective case for gene therapy for xerostomia.

## 9 Conclusion

Salivary gland gene therapy has several advantages. One of them is the stable population of salivary epithelial glands, which replicate slowly. This makes them a viable target for treatment. This type of therapy is also favorable because the relatively isolated nature of the salivary glands makes it highly unlikely for the potential vector to spread beyond them, and even if such a rare instance occurs, the salivary glands can be removed with minimal consequences [62, 64].

This review article acknowledges and consolidates the various contributions made by researchers to address the impairing condition of xerostomia; however, owing to the heterogeneous nature of this disease in terms of etiology as well as the limited potential of salivary glands to regenerate, it would not be amiss to view these developments in a skeptical light. As its effects range from affecting the patient's general health to deteriorating their quality of life, oral dryness cannot be written off as an inconsequential condition. There is a pressing need for innovative approaches that target conditions, considering their unique molecular mechanisms.

## Author Contributions

Conceptualization: Muhammad Usman Bilal and Hasan Mujtaba. Methodology: Muhammad Usman Bilal, Sarah Alvi, Fatima Humayune, Hasan Mujtaba, Shahzad Ahmad, and Muhammad Farooq Umer. Data curation: Muhammad Usman Bilal and Fatima Humayune. Investigation: Muhammad Usman Bilal, Sarah Alvi, Fatima Humayune, Hasan Mujtaba, Shazia Iqbal, and Muhammad Farooq Umer. Validation: Sarah Alvi, Fatima Humayune, Shahzad Ahmad, Shazia Iqbal, and Muhammad Farooq Umer. Formal analysis: Muhammad Usman Bilal. Supervision: Hasan Mujtaba. Writing – original draft preparation: Muhammad Usman Bilal, Sarah Alvi, Fatima Humayune, Hasan Mujtaba, Shahzad Ahmad, Shazia Iqbal, and Muhammad Farooq Umer. Writing – review and editing: Muhammad Usman Bilal, Sarah Alvi, Fatima Humayune, Hasan Mujtaba, Shahzad Ahmad, Shazia Iqbal, and Muhammad Farooq Umer. All authors have read and agreed to the published version of the manuscript.

## Conflicts of Interest

The authors declare no conflicts of interest.

## Transparency Statement

The corresponding authors, Hasan Mujtaba and Shahzad Ahmad, affirm that this manuscript is an honest, accurate, and transparent account of the study being reported; that no important aspects of the study have been omitted; and that any discrepancies from the study as planned (and, if relevant, registered) have been explained.

## Data Availability

The data will be provided on request by the corresponding author, Dr. Hasan Mujtaba.
